# Exaggerated Case of Purpura Hæmorrhagica of Twelve Months’ Standing Cured by Mercury

**Published:** 1879-01-20

**Authors:** John Herbert Claiborne

**Affiliations:** President of the Medical Society of Virginia, Etc.


### *EXAGGERATED CASE OF PURPURA HÆMORRHAGICA OF TWELVE MONTHS STANDING CURED BY MERCURY.*

BY JOHN HERBERT CLAIBORNE, M. D.,

*President of the Medical Society of Virginia, Etc.*

Mrs. Blank, æt. 34 years, mother of three children, youngest six years of age. She says that she has been suffering from purpura hæmorrhagica for twelve months; that it came on her suddenly after some slight indisposition—at first appearing on the lower limbs. Now her legs, arms, and the greater portion of her body are covered with spots of extravasated blood from the size of a lentil to that of a ten cent piece. There are no spots on the neck or face. She has emaciated but little, she says; weighs now some 150 pounds; has good complexion, rather florid than otherwise, but her muscular weakness is excessive, and she walks across the floor with the greatest difficulty. She is unable to go out at all, or to attend to any of her domestic duties. Has poor appetite, feeble digestion, and is very much annoyed with flatulence. Her bowels are irregular—disposed to constipation. The tongue is clean, and the mucous membrane of the digestive tract shows no evidence of being in any way affected by the disease. The kidneys are also healthy, and the uterine functions normal and regular. Her spirits are depressed to a degree which amounts almost to melancholy, and she expresses no hope of recovery. She has been treated by some half dozen physicians—some of eminence. All have agreed that the trouble was in the blood crasis, and have, without exception, exhibited iron and put her on full diet. Spent some two months with the late Dr. J. P. Mettauer, who also gave her iron, but used powerful astringent washes to the cuticle. On the supposition that the disease was one of indigestion, perhaps she was ordered to go to the Alleghany Springs, of this State, whose waters are famed as an alterative stomachic, but in no instance has treatment of any sort been of any avail. I immediately placed her on the following prescription:

℞. Hydrag. oxymuriat.....................grs. ij

Ext. cinchonæ.............................grs. xxiv.

M. Make pills, No. xxiv. S: One pill *ter die* after eating. Diet, liberal and nutritious, but unstimulating.

In one week's time the maculæ began to fade, and in one month had entirely disappeared. In the meantime the patient's strength had improved; her general health was almost restored, and she was enabled to attend to her ordinary duties. She has been well for some eighteen months, and though she has occasionally seen some evidence of a return of the purpura, it invariably and immediately retires before the use of her pills for a few days. There has never been the slightest ptyalism, though, on commencing the use of the pills, she took them for a month without suspending them for a day.

Mercury undoubtedly acted in this case, both as *alterative* and *tonic.* That it has a tonic action in certain pathological conditions of the system, I am sure. Fournier has called attention to this fact. Liegois has also remarked of it recently in some of his experiments, that "persons taking it, increased their weight," thus proving, that in some way, it acted as a stimulant or filip to the nutritive function. Wilbouchewitch, too, of Paris, by means of the hæmatametre counted daily the red corpuscles of persons under mercurial treatment, and found that they increased in number under the administration of that drug.

I was not led to try the use of mercury in this case, however, by any theoretical views, or on the report of the above distinguished authorities. The treatment *secundem artem recentem* had evidently failed, and I remembered that an old practitioner of physic—a man of great experience and great good sense—the late Dr. James May, had told me that he had several times succeeded in curing obstinate cases of purpura by the exhibition of mercury. He had no theory on the subject. *Virginia Medical Monthly, April,* 1878.

